# Vaginal Cuff Infection Caused by *Ureaplasma parvum* After Hysterectomy for Uterine Cervical Cancer: A Case Report

**DOI:** 10.1155/2024/4114954

**Published:** 2024-10-21

**Authors:** Hayato Chikamatsu, Mana Taki, Sachiko Kitamura, Masumi Sunada, Koji Yamanoi, Ryusuke Murakami, Ken Yamaguchi, Akihito Horie, Yasuhiro Tsuchido, Junzo Hamanishi, Masaki Mandai

**Affiliations:** ^1^Department of Gynecology and Obstetrics, Kyoto University Graduate School of Medicine, Kyoto, Japan; ^2^Department of Clinical Laboratory Medicine, Kyoto University Graduate School of Medicine, Kyoto, Japan

**Keywords:** hysterectomy, PCR, *Ureaplasma parvum*, vaginal cuff infection

## Abstract

*Ureaplasma parvum* is one of the most common endemic mycoplasmas in the genitourinary tract and can cause amniotic fluid infection leading to preterm labor. We report a rare case of *Ureaplasma parvum* infection ascending from the vagina to the abdominal cavity after hysterectomy, causing vaginal cuff infection, postoperative peritonitis, and small bowel obstruction. A 29-year-old nulliparous woman presented with infected uterine cervical cancer. After radical hysterectomy for uterine cervical cancer, the patient had paralytic ileus with ascites and fever. Peritonitis was suspected; however, all cultures were negative, making it difficult to identify the causative organism. Polymerase chain reaction (PCR) of the ascites revealed *Ureaplasma parvum*, which could be treated with levofloxacin (LVFX). Open drainage to control the infection revealed a necrotic tissue around vaginal cuff and the small intestine encased in cocoon-like fibers like sclerosing encapsulating peritonitis. After the infection was improved, the bowel obstruction was also improved. *Ureaplasma* spp. can be difficult to culture. PCR testing for *Ureaplasma* infection should be considered when urogenital infection is suspected in patients prone to opportunistic infections, such as those with malignant tumors.

## 1. Introduction

*Ureaplasma parvum* is one of the most common mycoplasmas endemic to the genitourinary tract [1]. It is an opportunistic infection that is frequently associated with infertility and premature birth [2, 3]. However, there have been no reports of vaginal cuff infection caused by *Ureaplasma* spp. infection following hysterectomy.

In this report, we describe a rare case of postoperative peritonitis caused by *Ureaplasma parvum* vaginal cuff infection after hysterectomy, in which ileus was the main symptom. The patient was difficult to treat because the causative organism could not be detected using conventional culture tests. In addition, the small intestine became adherent and encapsulated, resulting in sclerosing encapsulating peritonitis [4]. The identification of *Ureaplasma parvum* by polymerase chain reaction (PCR) test, switching to antibiotics that are effective against the microorganism, and open drainage led to the improvement in the infection and the bowel condition.

## 2. Case Report

**Figure 1:**
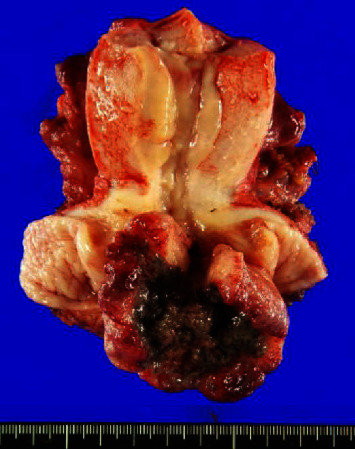
Specimen of the removed uterus. The cervical tumor has extensive central necrosis due to infection.

A 29-year-old nulliparous woman presented to our department complaining of cervical squamous cell carcinoma (SCC) with persistent genital bleeding, with a history of chlamydia infection ten years earlier. Purulent discharge was present, and infection of the cervical cancer was suspected. The patient was treated with clavulanic acid/amoxicillin for the infection, but there was no improvement. A radical hysterectomy was performed. There was no obvious peritoneal dissemination or adhesions in the abdominal cavity. The cervical tumor was infected with extensive necrosis (Figure 1). The final pathological diagnosis was cervical cancer SCC, pT1b3pN0MX, stage IB3 (FIGO 2018), without lymphovascular invasion.

**Figure 2:**
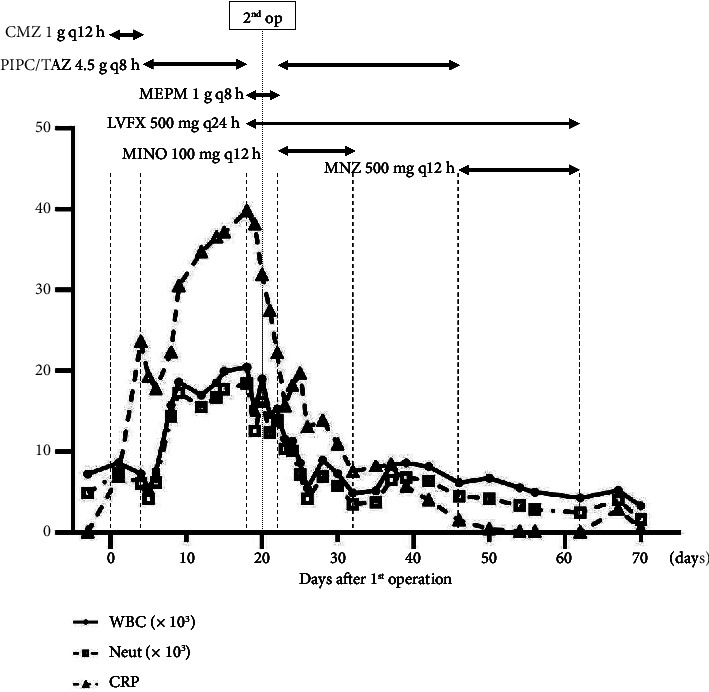
Postoperative clinical course of inflammatory response with antibiotic use.

**Figure 3:**
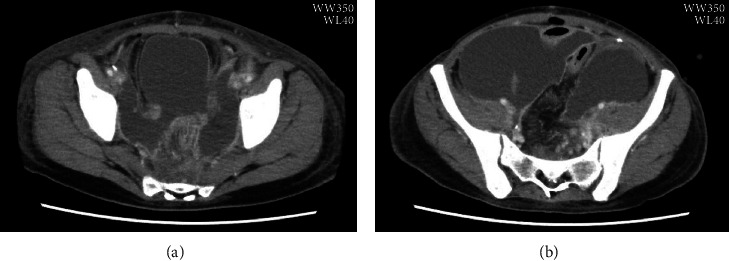
Computed tomography (CT) images on postoperative day 18. (a) Intestines are dilated, and the abdominal wall is thickened. Numerous septal walls can be seen in the abdominal cavity, where a large amount of ascitic fluid has accumulated. (b) The intestine is covered with a membranous capsule.

The patient had abdominal distention with a fever of 38°C postoperatively. Abdominal CT showed intestinal dilatation, which was thought to be paralytic ileus. The patient was treated conservatively with an ileal tube. The antibiotic was changed from cefmetazole at 1 g q12 h to tazobactam/piperacillin (TAZ/PIPC) at 4.5 g q8 h (Figure 2). The abdominal distention worsened, and ascites accumulated. The ileus continued, and peripheral parenteral nutrition was started on postoperative day 10, and total parenteral nutrition was started on postoperative day 17. The inflammatory response also worsened. On postoperative day 18, pelvic examination revealed white necrotic tissue adhering to the vaginal cuff. Repeated abdominal CT revealed that intestines were dilated, and the abdominal wall was thickened. There were septal walls in the abdominal cavity with a large amount of ascites (Figure 3). Peritonitis due to a vaginal cuff infection was suspected. The pus was drained from the vaginal cuff, though the culture was negative. Ascitic tap was performed, and the culture of ascites was also negative. However, PCR using VIASURE Sexually Transmitted Diseases Real Time PCR Detection Kit (Certest Biotec, Zaragoza, Spain) of the ascites detected *Ureaplasma parvum* and did not detect *Chlamydia trachomatis*, *Neisseria gonorrhoeae*, *Mycoplasma genitalium*, *Trichomonas vaginalis*, *Ureaplasma urealyticum*, and *Mycoplasma hominis*. The antibiotics were switched from TAZ/PIPC 4.5 g q8 h to meropenem (MEPM) 1 g q8 h and LVFX 500 mg q24 h. The inflammatory response gradually decreased. However, next day, her oxygen level was decreased. Since the infection was deemed poorly controlled, the patient underwent a second laparotomy for drainage.

**Figure 4:**
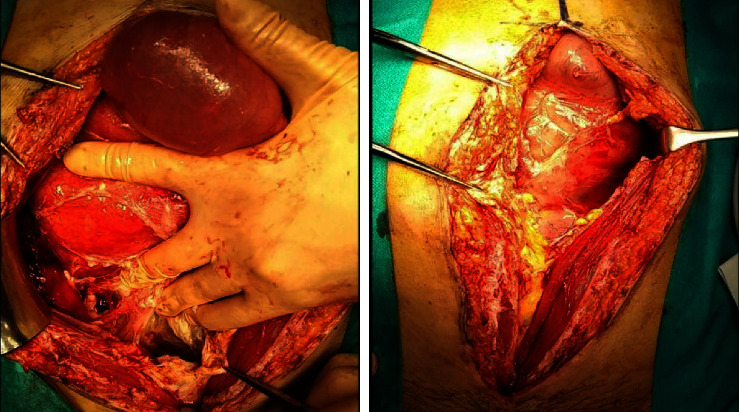
Intra-abdominal image. The small intestine is dilated and encased in cocoon-like fibrous membrane (sclerosing encapsulating peritonitis). It is firmly adherent to the abdominal wall. The right side of the vaginal cuff is covered with white mossy fibrosis due to infection.

There was white necrotic tissue around vaginal cuff and dense adhesions between peritoneum and intestinal tract. The small intestine was encased in a cocoon-like mass with a large amount of ascites like sclerosing encapsulating peritonitis (Figure 4). We removed necrotic tissue around vaginal cuff and cocoon-like mass around intestines and washed intraperitoneally.

The patient's general condition and inflammatory response gradually improved. Since her platelet count decreased to 35,000 on postoperative day 22, the antibiotic was switched from MEPM 1 g q8 h to PIPC/TAZ 4.5 g q8 h for suspected drug-induced thrombocytopenia. Minocycline (MINO) 100 mg was added every 12 h because *Ureaplasma parvum* may be resistant to LVFX. Her platelet counts quickly recovered to normal levels, and her inflammatory response, fever, and ileus were improved on postoperative day 27. On postoperative day 28, the ileus tube was removed and she resumed eating the following day. MINO was discontinued on postoperative day 33 and PIPC/TAZ was switched to metronidazole (MNZ) 500 mg q12 h on postoperative day 47, when her CRP level dropped to within the normal range. LVFX and MNZ were discontinued on postoperative day 63 in consideration of the possibility of a relapse (Figure 2).

## 3. Discussion

*Ureaplasma parvum*, a urogenital commensal bacterium, is the most common Mycoplasmataceae species isolated from the urogenital tract of both men and women [1]. It is considered an opportunistic pathogen that is often associated with infertility, preterm delivery, adverse pregnancy outcomes, and neonatal diseases such as chronic lung disease and retinopathy of prematurity [2, 3, 5, 6]. Cases of hyperammonemia and impaired consciousness due to septic arthritis have also been reported [7, 8]. It has also been reported to cause peritonitis associated with peritoneal dialysis [9]. In our case, the *Ureaplasma parvum* originally attached to the tumor invaded the abdominal cavity via vaginal cuff after hysterectomy.

Identifying *Ureaplasma parvum* as the causative agent of the infection is difficult because it does not grow in conventional bacterial cultures [10]. The development of genetic tests, such as PCR, will allow the rapid and sensitive diagnosis of *Ureaplasma parvum*. Treatment options for *Ureaplasma parvum* include antibiotics such as fluoroquinolones, doxycycline, clindamycin, and clarithromycin, whereas *β*-lactams, which target penicillin-binding proteins and inhibit cell-wall synthesis, are ineffective because *Ureaplasma parvum* has no cell wall. Furthermore, *Ureaplasma parvum* sometimes develops resistance to antibiotics, making it difficult to treat. *Ureaplasma* species are generally susceptible to fluoroquinolones, macrolides, and tetracyclines; however, resistance of these antibiotics has been reported [11, 12].

At second laparotomy for drainage, intestines were encapsuled in fibrotic tissue forming cocoon-like mass like sclerosing encapsulating peritonitis. Sclerosing encapsulating peritonitis, first described in 1978, refers to the complete or partial encasement of the small intestine by a thick, cocoon-like fibrous membrane that causes acute or chronic intestinal obstruction and/or an abdominal mass [4, 13, 14]. Sclerosing encapsulating peritonitis is classified as idiopathic or secondary [15]. The idiopathic sclerosing encapsulating peritonitis is mainly observed in tropical and subtropical regions and the etiology is unknown. Although some hypothesize retrograde menstruation superimposed on viral infection or cell-mediated immune tissue damage secondary to gynecological infection, some believe that this disorder, which is twice as common in males as in females, is the result of a developmental disorder citing vascular abnormalities and omental hypoplasia [15–17]. Secondary sclerosing encapsulating peritonitis, which is thought to be caused by peritoneal dialysis and recurrent peritonitis, is more common than idiopathic one. Some cytokines release an intraperitoneal fibrin that forms a thick, shiny membrane that envelops all or parts of the intestine. In our case, it probably developed from vaginal cuff infection by *Ureaplasma parvum*, leading to the small bowel obstruction. In addition, the patient's condition did not improve with antibiotics alone, and surgical drainage was required. This is thought to be due to the abscess at the vaginal cuff as well as sclerosing encapsulating peritonitis, which caused the ascites to be divided by septal walls.

## 4. Conclusions

We encountered a case of *Ureaplasma parvum* vaginal cuff infection that developed because of surgery, leading to bowel obstruction. As conventional culture tests cannot detect this organism, PCR should be considered when urogenital infection is suspected in immunocompromised patients, such as those with malignancy.

## Data Availability

Data supporting this research article are available from the corresponding author on reasonable request.
